# Combining multiple ECG features does not improve prediction of defibrillation outcome compared to single features in a large population of out-of-hospital cardiac arrests

**DOI:** 10.1186/s13054-015-1142-z

**Published:** 2015-12-10

**Authors:** Mi He, Yushun Gong, Yongqin Li, Tommaso Mauri, Francesca Fumagalli, Marcella Bozzola, Giancarlo Cesana, Roberto Latini, Antonio Pesenti, Giuseppe Ristagno

**Affiliations:** School of Biomedical Engineering, Third Military Medical University and Chongqing University, 30 Gaotanyan Main Street, Chongqing, 400038 China; Department of Anesthesia, Critical Care and Emergency, Fondazione IRCCS Ca’ Granda Ospedale Maggiore Policlinico, Via Francesco Sforza, 35, 20122 Milan, Italy; IRCCS-Istituto di Ricerche Farmacologiche “Mario Negri”, Via Privata Giuseppe La Masa, 19, 20156 Milan, Italy; Azienda Regionale Emergenza Urgenza (AREU), Via Alfredo Campanini, 6, 20124 Milan, Italy; Research Centre on Public Health, Department of Statistics and Quantitative Methods, University of Milano-Bicocca, Piazza dell’Ateneo Nuovo, 1, 20126 Milan, Italy

**Keywords:** Defibrillation, Ventricular fibrillation, Predictive features, Combination, Cardiac arrest

## Abstract

**Introduction:**

Quantitative electrocardiographic (ECG) waveform analysis provides a noninvasive reflection of the metabolic milieu of the myocardium during resuscitation and is a potentially useful tool to optimize the defibrillation strategy. However, whether combining multiple ECG features can improve the capability of defibrillation outcome prediction in comparison to single feature analysis is still uncertain.

**Methods:**

A total of 3828 defibrillations from 1617 patients who experienced out-of-hospital cardiac arrest were analyzed. A 2.048-s ECG trace prior to each defibrillation without chest compressions was used for the analysis. Sixteen predictive features were optimized through the training dataset that included 2447 shocks from 1050 patients. Logistic regression, neural network and support vector machine were used to combine multiple features for the prediction of defibrillation outcome. Performance between single and combined predictive features were compared by area under receiver operating characteristic curve (AUC), sensitivity, specificity, positive predictive value (PPV), negative predictive value (NPV), and prediction accuracy (PA) on a validation dataset that consisted of 1381 shocks from 567 patients.

**Results:**

Among the single features, mean slope (MS) outperformed other methods with an AUC of 0.876. Combination of complementary features using neural network resulted in the highest AUC of 0.874 among the multifeature-based methods. Compared to MS, no statistical difference was observed in AUC, sensitivity, specificity, PPV, NPV and PA when multiple features were considered.

**Conclusions:**

In this large dataset, the amplitude-related features achieved better defibrillation outcome prediction capability than other features. Combinations of multiple electrical features did not further improve prediction performance.

## Introduction

Early cardiopulmonary resuscitation (CPR) and early defibrillation are the key points in the chain of survival in cardiac arrest patients with shockable rhythms [1, 2]. However, the priority of intervention, CPR or immediate defibrillation and the duration of CPR intervals prior to defibrillation are still debated, particularly in out-of-hospital cardiac arrests (OHCA) with long response times [3–5]. Animal studies demonstrated that high success of restoration of spontaneous circulation (ROSC) is achieved when the heart is recently perfused, while prolonged untreated ventricular fibrillation (VF) with depleted energy phosphates leads to poor outcome [6]. Clinical studies also indicated that not all VF patients benefit from being treated in the same manner with a time-based CPR/defibrillation protocols [2, 7]. Optimizing timing of defibrillation might decrease the severity of postresuscitation myocardial dysfunction by reducing the numbers of failed shocks and by reducing the consequent unnecessary interruptions in chest compression, having therefore the potential to improve the final outcome of cardiac arrest [8].

Quantitative electrocardiogram (ECG) waveform analysis provides a noninvasive reflection of the metabolic status of the myocardium during resuscitation and is a potential tool to guide and optimize CPR interventions, i.e., chest compression or defibrillation [9]. During the last two decades, numerous features have been developed and used to predict the outcome of defibrillation, including time domain [10–15], frequency domain [15–19] and nonlinear measures [20, 22]. As combining multiple predictive features may offer complementary information to improve the predictive accuracy [16], several studies have been attempted to combine different VF features to enhance the predictive performance using the machine learning theory, albeit in relatively small populations [25, 25]. Whether the combination of multiple predictive features can improve prediction capability for defibrillation outcome compared to the single features is still uncertain.

The purpose of the present study was to investigate whether combination of multiple VF features, by different machine learning strategies, including logistical regression (LR), artificial neural network and support vector machine (SVM), could improve the prediction capacity of defibrillation outcome using a large multicenter database of OHCA patients.

## Methods

### Data sources

This study was approved by the ethics committee of the coordinating center, San Gerardo University Hospital, Monza, Italy. The institutional review board waived the requirement of informed consent since the data were already collected for administrative and statistical reasons by the National Health System.

A total of 3828 defibrillation shocks from 1617 patients who experienced OHCA were analyzed. The detailed descriptions of the multicenter database and population characteristics have been previously reported [18]. Data included a training set of 2447 defibrillations from 1050 patients and a validation set of 1381 defibrillations from 567 patients. All ECG data were digitally resampled at 250 Hz for compatibility with other studies. A 2.048-s episode (512 samples) free from chest compression was selected immediately prior to each defibrillation. Preprocessing of ECG data was executed by bandpass filters with different frequency ranges for baseline drifting removal and artifact attenuation.

Successful defibrillation was defined as the achievement of an organized rhythm with heart rate ≥ 40 beats/min within 60 s postdefibrillation, while shocks resulting in VF, ventricular tachycardia (VT), asystole or pulseless electrical activity with pauses > 3 s were regarded as unsuccessful defibrillations [18]. In the training set, 641 defibrillations (26.2 %) were successful, while considering only the 1050 first defibrillation attempts, 278 (26.5 %) were successful. In the validation set, 445 defibrillations (32.1 %) were successful and 175 (31.0 %) were successful when only the first defibrillations were considered.

### Predictive feature selection and optimization

Sixteen predictive features of ECG waveforms with good prediction power in previous clinical studies [12, 26] were selected and calculated in this study. Table 1 presents their definitions and equations.

**Table 1:** Predictive features and their calculations

Category	Predictive feature	Equation
Time domain	Mean slope (MS)	$\frac{f_s}{N-1}{\displaystyle \sum_{i=2}^N\left|{x}_i-{x}_{i-1}\right|}$
	Median slope (MdS)	median(*x*_*i*_ − *x*_*i* − 1_)*f*_*s*_
	Amplitude range (AR)	max(*x*_*i*_) − min(*x*_*i*_)
	Signal integral (SignInt)	${\displaystyle \sum_{i=1}^N\left|{x}_i\right|}$
	Average peak-to-peak amplitude (PPA)	$\frac{1}{L}{\displaystyle \sum_{i=1}^L\left( \max \left({x}_{i,L}\right)- \min \left({x}_{i,L}\right)\right)}$
	Root mean square (RMS)	$\sqrt{\frac{1}{N}{\displaystyle \sum_{i=1}^N{\left({x}_i-\overline{x}\right)}^2}}$
Frequency domain	Amplitude spectral area (AMSA)	${\displaystyle \sum_{j=1}^M{A}_j{f}_j}$
	Power spectrum analysis (PSA)	${\displaystyle \sum_j{P}_x\left({f}_j\right)\cdot {f}_j}$
	Max power (MP)	$\underset{j}{ \max}\left({P}_x\left({f}_j\right)\right)$
	Peak frequency (PF) or dominant frequency	$\underset{j}{ \arg \max}\left({P}_x\left({f}_j\right)\right)$
	Centroid frequency (CF)	$\frac{{\displaystyle \sum_j{f}_j\cdot {P}_x\left({f}_j\right)}}{{\displaystyle \sum_j{P}_x\left({f}_j\right)}}$
	Energy (EG)	${\displaystyle \sum_j{P}_x\left({f}_j\right)}$
Others	Spectral flatness measure (SFM)	$\frac{ \exp \left(\frac{1}{M}{\displaystyle \sum_j \ln \left({P}_x\left({f}_j\right)\right)}\right)}{\frac{1}{M}{\displaystyle \sum_j{P}_x\left({f}_j\right)}}$
	Wavelet energy (WE)	${\displaystyle \sum_j\left|{W}_x\left({c}_j\right)\right|}$
	Spectrum entropy (SPE)	$\hbox{-} {\displaystyle \sum_{i=1}^L\left[\left(\frac{{\displaystyle \sum_{f_{j,L}}{P}_x\left({f}_{j,L}\right)}}{{\displaystyle \sum_j{P}_x\left({f}_j\right)}}\right)\cdot { \log}_2\left(\frac{{\displaystyle \sum_{f_{j,L}}{P}_x\left({f}_{j,L}\right)}}{{\displaystyle \sum_j{P}_x\left({f}_j\right)}}\right)\right]}$
	Hurst index (Hu)	$\frac{ \log \left[R(i)/S(i)\right]}{ \log (i)}$

*x*_*i*_ (*i* = 1,2,…, *N*) represented samples of ECG segment *x*(*t*) in time domain with sampling rate *f*_*s*_, and mean value $\overline{x}$. *A*_*j*_ indicated the amplitude of Fourier transform of *x*(*t*) at frequency *f*_*j*_ (*j* = 1,2,…, *M*). *P*_*x*_(*f*_*j*_) specified samples power spectral density of *x*(*t*) at frequency *f*_*j*_. *W*_*x*_(*c*_*j*_) represented samples of high-band coefficients of wavelet transform of *x*(*t*). *L* in PPA indicated *L* subintervals; *L* in SPE indicated *L* frequency bands. Function *R*(·) was taken as the difference between the maximum and minimum deviation from time period "*i*". Function *S*(·) calculated the standard deviation for time period "*i*"

Optimum frequency range of bandpass filters for each feature was obtained with a criterion of maximum area under the receiver operating characteristic (ROC) curve (AUC) using the training data. The boundaries of the lower and upper frequencies for calculating the optimum frequency range were 2–5 Hz and 20–48 Hz, respectively.

### Combination methods

Three different machine learning techniques, including LR, neural network and SVM were used to combine different VF features for the prediction of defibrillation outcome.

#### Logistic regression

In the LR model, optimal features (with *p* values all less than 0.0001) were automatically selected from the 16 features employing the training data by forward stepwise using the likelihood ratio test. The LR equation for prediction was $\frac{1}{1+ \exp \left(-{\beta}_0-{\displaystyle \sum_n{\beta}_n{y}_n}\right)}$, where *β*_0_ is the regression constant and *β*_*n*_ is the nth regression coefficient of the selected feature *y*_*n*_. The predictive values of validation data were obtained according to the corresponding LR equations by a threshold (for successful or unsuccessful decisions) with equal sensitivity and specificity for the training data.

#### Neural network

The back propagation (BP) neural network with a feed forward structure was used in the training set to achieve an optimal outcome. The training processing adopted the Bayesian regularization training function, two hidden layers, and sigmoid and linear transfer functions. All features in the training and validation sets were normalized by minus of mean and division of standard deviation values. The AUC of direct outcomes of the BP neural network was calculated as these outcomes were not binary decisions (0 or 1). For compatibility, a threshold with equal sensitivity and specificity for training data was used to result in a binary decision. Combination of all features (BP-C1), combination of features with a high predictive power (with AUC > 0.8) (BP-C2) and combination of complementary features (correlation coefficient r < 0.3) (BP-C3) were tested by the BP neural network, respectively.

#### Support vector machine

In the SVM model, a Gaussian radial basis function was selected as the kernel function with an error penalty factor (C = 1) and a scaling factor (σ = 0.01). Choosing small values for the error penalty factor and the scaling factor was intended to make the risk function of SVM have solutions for large training data. Combinations of all features (SVM-C1), high predictive power features (SVM-C2) and complementary features (SVM-C3) were also adopted in the training and validation processes of SVM.

### Statistical analysis

The prediction power was assessed by ROC curves, AUC, sensitivity, specificity, positive predictive value (PPV), negative predictive value (NPV) and prediction accuracy (PA) [18, 26]. For compatibility with the machine learning techniques, sensitivity, specificity, PPV, NPV and PA of single features for the validation data were calculated with a threshold in which sensitivity equaled to specificity for the training data.

Pearson's correlation coefficients were calculated among single features for correlation analysis. AUCs were compared using Z-test. Chi-squared test was employed to distinguish differences among sensitivity, specificity, PPV, NPV and PA of the different predictive features. A final two-tailed *p* value < 0.05 was considered statistically significant.

## Results

### Performance of single features

**Fig. 1:**
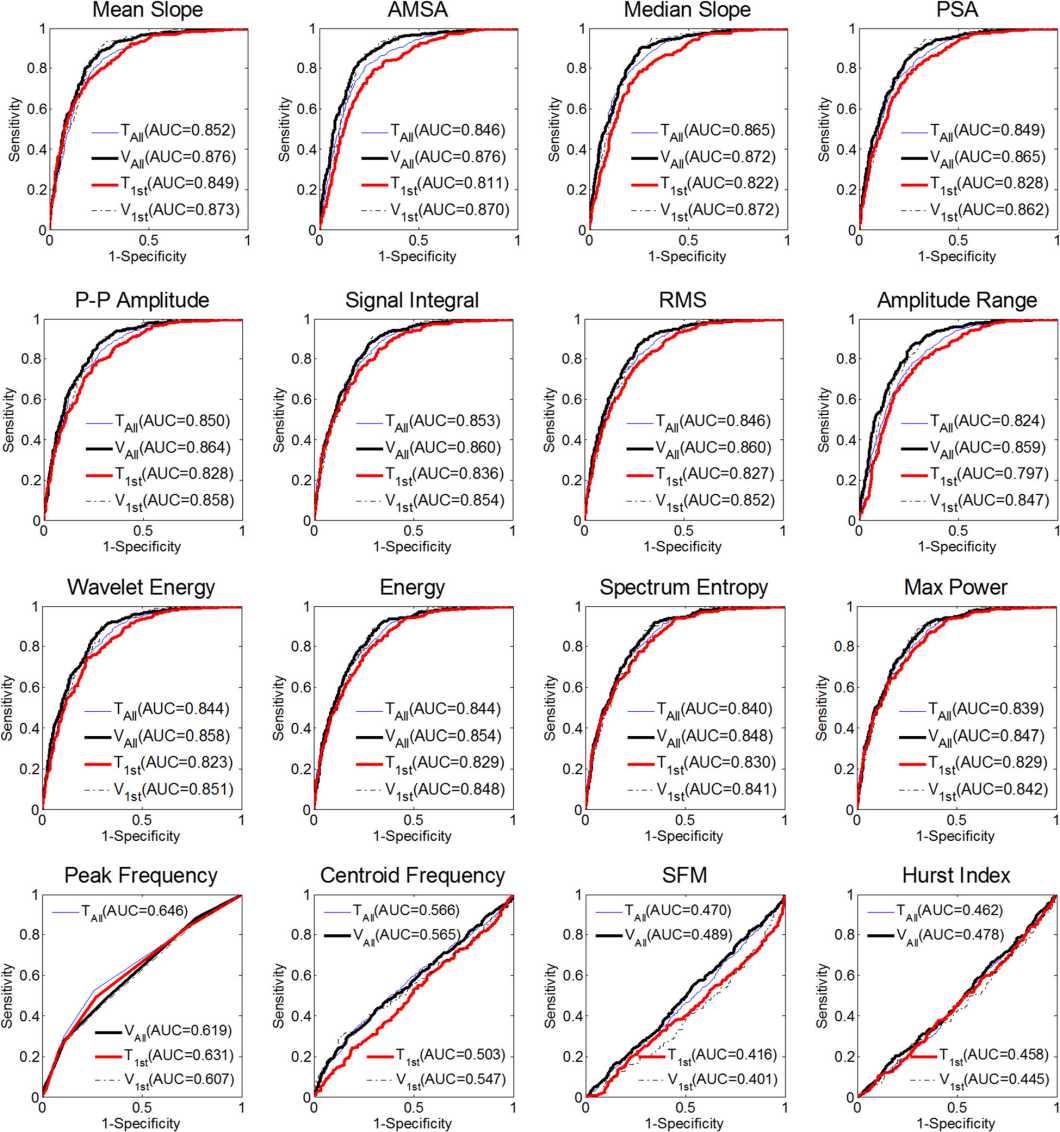
Receiver operating characteristic (ROC) curves and area under ROC curves (AUC) of 16 the predictive features for training and validation datasets. *1st* first defibrillations, *All* all defibrillations, *AMSA* amplitude spectrum analysis, *P-P amplitude* average peak-peak amplitude, *RMS* root mean square, *SFM* spectral flatness measure, *T* training set, *V* validation set

ROC curves and AUCs of the candidate features for all and the first defibrillations in training and validation datasets are reported in Fig. 1. All the 16 candidate VF features, except for peak frequency (PF), centroid frequency (CF), spectral flatness measure (SFM), and Hurst index (Hu), showed a high AUC, i.e., > 0.8. More specifically, mean slope (MS) and amplitude spectral area (AMSA) had the highest AUC values (0.876) for all defibrillations, while MS had the highest AUC value (0.873) for the first defibrillations in the validation set. Median slope (MdS), power spectrum analysis (PSA), average peak-to-peak amplitude (PPA), signal integral (SignInt), root mean square (RMS), amplitude range (AR), wavelet energy (WE) and energy (EG) also had an AUC value greater than 0.845 (*p* was not significant vs. MS for all and/or for the first defibrillations). Considering all the defibrillation attempts, AUCs for spectrum entropy (0.848, *p* = 0.024 vs. MS) and max power (0.847, *p* = 0.020 vs. MS) were relatively lower when compared with MS, but no significant differences were observed when the first defibrillations were considered. Additionally, AUCs for PF (0.619/0.607), CF (0.565/0.547), SFM (0.489/0.401) and Hu (0.478/0.445) were significantly lower compared with MS (*p* < 0.001), both for all and first defibrillations.

Correlation analysis demonstrated that most of the features were significantly correlated with each other (Table 2). Amplitude-related features, such as MS, AMSA, MdS, SignInt, PSA, PPA, WE, AR and RMS were strongly correlated with each other (r > 0.807, *p* < 0.001). For frequency-related methods, CF was highly correlated with PF (r = 0.770, *p* < 0.001) and SFM (r = 0.829, *p* < 0.001). Poor correlations were observed among the other measures.

**Table 2:** Correlation coefficients among the 16 candidate features used for defibrillation outcome prediction

	MS	AMSA	SignInt	PSA	PPA	Mds	MP	PF	CF	EG	SFM	WE	AR	SPE	RMS
AMSA	0.86**														
SignInt	0.81**	0.91**													
PSA	0.93**	0.84**	0.90**												
PPA	0.85**	0.94**	0.98**	0.91**											
Mds	0.85**	0.91**	0.93**	0.86**	0.92**										
MP	0.31**	0.14**	0.18**	0.36**	0.26**	0.16**									
PF	0.20**	0.28**	0.34**	0.24**	0.34**	0.32**	0								
CF	0.08**	0.17**	0.15**	0.09**	0.17**	0.10**	-0.08**	0.77**							
EG	0.14**	-0.02	-0.01	0.17**	0.07**	-0.01	0.98**	-0.05**	-0.10**						
SFM	-0.04	0	-0.12**	-0.07**	-0.06**	-0.16**	0.03	0.43**	0.83**	0.07**					
WE	0.86**	0.82**	0.92**	0.95**	0.90**	0.84**	0.21**	0.28**	0.14**	0.01	-0.06**				
AR	0.81**	0.93**	0.91**	0.85**	0.94**	0.82**	0.21**	0.30**	0.19**	0.04*	0.02	0.86**			
SPE	0.07**	0.13**	0.12**	0.07**	0.12**	0.12**	0.02	0.06**	0	0	-0.07**	0.07**	0.12**		
RMS	0.83**	0.88**	0.95**	0.91**	0.97**	0.88**	0.46**	0.31**	0.12**	0.29**	-0.07**	0.88**	0.92**	0.11**	
Hu	0	0.05*	-0.04	-0.01	0	-0.11**	0.04	-0.10**	-0.02	0.04*	0.10**	0	0.15**	0	0.02

*MS* mean slope, *AMSA* amplitude spectral area, *SignInt* signal integral, *PSA* power spectrum analysis, *PPA* average peak-to-peak amplitude, *Mds* median slope, *MP* max power, *PF* peak frequency, *CF* centroid frequency, *EG* energy, *SFM* spectral flatness measure, *WE* wavelet energy, *AR* amplitude range, SPE spectrum entropy, *RMS* root mean square, *Hu* Hurst index

*p < 0.05, **p < 0.01

### Performance of combined features

The performance of combined features in the validation set for all and first defibrillations are listed in Tables 3 and 4, respectively.

**Table 3:** Prediction power of combination methods and single features for all defibrillations in the validation dataset (445 successful shocks/1381 shocks)

Methods	AUC	Sensitivity (%)	Specificity (%)	NPV (%)	PPV (%)	PA (%)
LR	0.872	79.6	79.6	89.1	65.0	79.6
BP-C1	0.873	80.5	80.5	89.7	66.2	80.5
BP-C2	0.873	80.0	80.4	89.4	65.6	80.3
BP-C3	0.875	80.9	80.9	89.9	66.8	80.9
SVM-C1	N/A	N/A	67.8	100.0	0.0	67.8
SVM-C2	N/A	N/A	67.8	100.0	0.0	67.8
SVM-C3	N/A	71.3	80.1	89.9	53.0	78.0
MS	0.876	78.0	82.9	88.8	68.4	81.3
AMSA	0.876	79.6	81.4	89.3	67.0	80.8
MdS	0.872	79.8	80.9	89.4	66.5	80.5

C1, C2 and C3 represented combination of all features, combination of features with a high predictive power (AUC > 0.8) and combination of complementary features (MS and SFM) using BP neural network, respectively

*AUC* area under receiver operating characteristic curve, *NPV* negative predictive value, *PPV* positive predictive value, *PA* prediction accuracy, *LR* logistic regression method, *BP* back propagation neural network method, *SVM* support vector machine method, *MS* mean slope, *AMSA* amplitude spectral area, *Mds* median slope, *N/A* not existing

**Table 4:** Prediction power of combination methods and single features for the first defibrillations in the validation data (175 successful shocks/567 shocks)

Methods	AUC	Sensitivity (%)	Specificity (%)	NPV (%)	PPV (%)	PA (%)
LR	0.870	79.6	79.6	89.1	65.0	79.6
BP-C1	0.864	78.9	78.9	89.2	62.4	78.7
BP-C2	0.868	80.0	79.9	89.9	64.2	80.0
BP-C3	0.873	80.0	80.0	89.9	64.2	80.0
SVM-C1	N/A	N/A	69.0	100.0	0.0	69.0
SVM-C2	N/A	N/A	69.0	100.0	0.0	69.0
SVM-C3	N/A	67.0	76.2	92.0	36.0	74.6
MS	0.873	84.0	79.2	91.7	64.5	80.7
AMSA	0.870	73.1	82.8	87.3	65.6	79.8
MdS	0.872	76.0	82.0	88.4	65.5	80.1

C1, C2 and C3 represented combination of all features, combination of features with a high predictive power (AUC > 0.8) and combination of complementary features (MS and SFM) using BP neural network, respectively

*AUC* area under receiver operating characteristic curve, *NPV* negative predictive value, *PPV* positive predictive value, *PA* prediction accuracy, *LR* logistic regression method, *BP* back propagation neural network method, *SVM* support vector machine method, *MS* mean slope, *AMSA* amplitude spectral area, *Mds* median slope, *N/A* not existing

Combining MS and SFM with BP neural network (BP-C3) resulted in the highest AUC (0.875/0.873) and accuracy (80.9 %/80.0 %) for all and first defibrillations, but no statistical differences were observed when compared with the combined LR, BP-C1 and BP-C2, for all and first defibrillations. Compared with SVM-C3, BP-C3 predicted outcome of all defibrillations with higher sensitivity (80.9 % vs. 71.3 %, *p* < 0.001), specificity (80.9 % vs. 80.1 %, *p* < 0.001) and NPV (66.8 % vs. 53.0 %, *p* < 0.001). It also showed higher sensitivity (80.0 % vs. 67.0 %, *p* = 0.015), PPV (64.2 % vs. 36.0 %, *p* < 0.001) and PA (80.0 % vs. 74.6 %, *p* = 0.033) compared to SVM-C3 when the first defibrillations were considered.

### Comparison between single and combined features with optimal performance

Since BP-C3 outperformed other combination strategies and MS had optimal performance among single feature methods, the prediction capacity between MS and BP-C3 was then compared. There were no statistical differences in AUC (*p* = 0.471 and 0.444), sensitivity (*p* = 0.281 and 0.330), specificity (*p* = 0.254 and 0.790), PPV (*p* = 0.568 and 0.955), NPV (*p* = 0.453 and 0.422) and PA (*p* = 0.771 and 0.765) between BP-C3 and MS for all (Table 3) and the first (Table 4) defibrillations.

## Discussion

In the present study, we investigated whether combination of multiple VF features could improve the capability of defibrillation outcome prediction using a large multicenter database from OHCA patients by machine learning strategies. The results indicated that the amplitude-related features outperformed other single waveform measures, while combining multiple VF features did not further improve the capability of defibrillation prediction.

Accuracy in predicting defibrillation outcome during resuscitation of VF cardiac arrest patients provides the potential to significantly enhance resuscitative strategies and improve patient’s outcome. A considerable number of defibrillation predictors have been proposed and shown to be promising in estimating VF duration, predicting defibrillation outcome, return to organized rhythm, and prognosticating long-term survival [10–23]. Current best predictors achieve an AUC in predicting defibrillation outcome of 0.87, with a balanced sensitivity and specificity of approximately 80 %. The above approaches have already a high predictive power; nevertheless research identifying approaches that might further improve the accuracy of defibrillation outcome prediction for OHCA is still ongoing. A possible solution is to use patient-specific information in the ECG-based prediction model. In an earlier study, Monsieurs et al. showed that adding age to the prediction formula increased the correct classification of survivors and nonsurvivors in 100 OHCA victims [27]. However, no significant improvement was obtained by including age, sex, presenting rhythm, presence of bystander CPR and ambulance response time when six different single prediction features were investigated in 530 shocks from 86 patients [28].

Another practical approach to improve the predictive performance of current ECG analysis is to combine multiple VF features using machine learning strategies. In a dataset of 883 defibrillations from 156 OHCA patients, Eftestøl et al. demonstrated that the combination of two decorrelated spectral features based on the principal component analysis of an original feature dataset with information on CF and PF could reduce the number of unsuccessful defibrillations [29]. In another database including 203 defibrillations from 47 patients with OHCA, Podbregar et al. reported that combining VF features including maximal amplitude, total energy of power spectral density and the Hurst exponent by genetic programming could potentially reduce the incidence of unsuccessful defibrillations [23]. On the contrary, Watson et al. showed no improvement in defibrillation outcome prediction performance when combined entropy with four other features in comparison with the five wavelet-based features alone [30]. In another clinical study, Neurauter et al. compared the performance of ten single predictive features and their combinations in 770 countershock attempts from 197 patients, and verified that combination of these predictive features using neural networks could not improve outcome prediction [25]. Recently, Shandilya et al. predicted defibrillation success using a parametrically optimized SVM model from a database of 90 precountershock ECG signals. The PA (82.2 % vs. 64.6 %) and AUC (0.850 vs. 0.609) were considerably improved by combining six to ten features compared with single feature-based AMSA [22]. Howe et al. investigated an alternative SVM-optimized classification approach, which combined multiple metrics with acceptable predictive attributes in a total of 115 defibrillations from 41 patients [24]. In contrast to the 86 % sensitivity and 60 % specificity for single feature AMSA, performance of the combined features was improved to a sensitivity of 87.6 % and a specificity of 71.6 % for the prediction of return of organized rhythm.

Besides the differences in machine learning methods and feature selection [22–25], the relative smaller sample size and not multicenter data might be responsible for the controversial conclusions when multiple features were applied to predict defibrillation outcome. In previous clinical studies, data were usually split into training and validation sets to testify the performance of predictors or designed parameters [17, 18, 22–25]. Switching the role of two sets by a crossvalidation method was frequently adopted to increase the degree of expected reliability in studies with relative smaller sample size [22–25]. Nevertheless, the test performances were considered in the design of the classifiers to optimize and generalize parameters [17]. Thereby, the crossvalidation strategy would influence the design process and bias the validation results.

Our results, obtained from the largest database of ECG traces on OHCA patients to date, showed that amplitude-related measures, such as MS, AMSA, MdS, PSA, PPA outperformed frequency and nonlinear-based methods when ranked by AUC and exhibited similar shock success prediction performance, consistent with the study of Wu and Firoozabadi et al [14, 26]. However, combining multiple VF features did not further improve the capability of defibrillation prediction in comparison to single features. This result was consistent with Neurauter et al. [25] when neural network was used but was controversial to the study of Howe et al. [24] when SVM was applied to combine multiple features. Notably, limited clinical data (115 defibrillations from 41 patients) were used in a crossvalidation SVM approach by Howe et al. [24], which might have caused biased validation results. Moreover, SVM usually keeps a desirable predictive performance for a small number of samples, but a large number of samples with noise may cause overfitting and overspecialization during the training process of SVM and create a negative bias in accuracies when the validation data are passed through the model [31]. Though overfitting happened when using the neural network with multiple hidden layers as well, neural network seemed more robust than SVM for a large number of training samples, which was caused by the different optimization functions and output variable forms employed in these two machine learning methods [31].

The unimproved prediction power of multiple VF features may be due to the limited information obtained from ECG signals and indicates that various single VF features, such as MS and AMSA, already reached the maximum prediction power extractable from VF ECGs. Besides ECG waveform characteristics, outcome of defibrillation is related to other factors of patients, such as drug treatments, comorbidities, and Emergency Medical Systems (EMS) arrival time. Additional clinically relevant attributes, independent from ECG waveform metrics, such as end-tidal carbon dioxide, blood pressure, blood oxygen saturation and compression depths, might be considered to further improve prediction power [22]. From another point of view, the longitudinal ECG data often has repeated defibrillations on each patient. The treatment effects and relative changes of a certain predictive feature may enhance the prediction performance in some degree.

We recognized that several limitations need to be considered in the study. First, this was a retrospective study on prospectively collected data. Sixteen predictive features were calculated only during the predefibrillation hands-off time and not in real time during chest compression. Second, the successful defibrillation was defined as sustained ROSC, but long-term survival was not considered. Peri-arrest factors such as age, sex, presenting rhythm, EMS arrival time, drug treatments, comorbidities, were not analyzed in this study. Third, further studies including independent ECG waveform metrics should to be tested in future prospective evaluations.

## Conclusions

In this large population of OHCA patients, amplitude-related features such as MS, AMSA and MdS, achieved better prediction power of defibrillation outcome than other features. Combining multiple electrical features did not further improve prediction performance in comparison to the single features.

## Key messages

- The electrical features obtained from ECG waveform are promising in prediction of defibrillation outcome. However, most of these features are highly correlated with each other.
- The amplitude related features achieve better defibrillation outcome prediction capability than other features.
- Combinations of multiple electrical features using machine learning strategies does not further improve prediction performance.

## Abbreviations

AMSA: amplitude spectral area
AR: amplitude range
AUC: Area under the ROC curve
BP: Back propagation neural network method
CF: Centroid frequency
EG: Energy
Hu: Hurst index
LR: logistic regression method
MdS: Median slope
MS: Mean slope
PSA: Power spectrum analysis
PPV: Positive predictive value
RMS: Root mean square
ROC: Receiver operating characteristic
ROSC: Return of spontaneous circulation
SFM: Spectral flatness measure
SignInt: Signal integral
SVM: Support vector machine method
WE: Wavelet energy
VF: Ventricular fibrillation
VT: ventricular tachycardia
